# A dual-scale fused hypergraph convolution-based hyperedge prediction model for predicting missing reactions in genome-scale metabolic networks

**DOI:** 10.1093/bib/bbae383

**Published:** 2024-08-05

**Authors:** Weihong Huang, Feng Yang, Qiang Zhang, Juan Liu

**Affiliations:** Institute of Artificial Intelligence, School of Computer Science, Wuhan University, Wuhan, Hubei 430072, China; Institute of Artificial Intelligence, School of Computer Science, Wuhan University, Wuhan, Hubei 430072, China; Institute of Artificial Intelligence, School of Computer Science, Wuhan University, Wuhan, Hubei 430072, China; Institute of Artificial Intelligence, School of Computer Science, Wuhan University, Wuhan, Hubei 430072, China

**Keywords:** missing reaction, genome-scale metabolic model, hyperedge prediction, dual-scale fused hypergraph convolution

## Abstract

Genome-scale metabolic models (GEMs) are powerful tools for predicting cellular metabolic and physiological states. However, there are still missing reactions in GEMs due to incomplete knowledge. Recent gaps filling methods suggest directly predicting missing responses without relying on phenotypic data. However, they do not differentiate between substrates and products when constructing the prediction models, which affects the predictive performance of the models. In this paper, we propose a hyperedge prediction model that distinguishes substrates and products based on dual-scale fused hypergraph convolution, DSHCNet, for inferring the missing reactions to effectively fill gaps in the GEM. First, we model each hyperedge as a heterogeneous complete graph and then decompose it into three subgraphs at both homogeneous and heterogeneous scales. Then we design two graph convolution-based models to, respectively, extract features of the vertices in two scales, which are then fused via the attention mechanism. Finally, the features of all vertices are further pooled to generate the representative feature of the hyperedge. The strategy of graph decomposition in DSHCNet enables the vertices to engage in message passing independently at both scales, thereby enhancing the capability of information propagation and making the obtained product and substrate features more distinguishable. The experimental results show that the average recovery rate of missing reactions obtained by DSHCNet is at least 11.7% higher than that of the state-of-the-art methods, and that the gap-filled GEMs based on our DSHCNet model achieve the best prediction performance, demonstrating the superiority of our method.

## Introduction

The genome-scale metabolic model (GEM) plays a crucial role in the fields of biology and bioengineering, revealing the metabolic processes within cells and throughout entire organisms. It usually serves as a mathematical expression of an organism’s metabolic capabilities and stands as a powerful computational tool for predicting metabolic fluxes, which is primarily inferred from genome annotations, unveil the intricate details of cellular and organism-wide metabolism. The GEMs demonstrate significant practicality in synthetic biology, metabolic engineering, and systems medicine [1–4]. The rapid growth in whole-genome sequencing data has led to a surge in draft GEMs generated by automatic reconstruction pipelines [5–7]. However, there are still numerous knowledge gaps remained within these models, including unannotated and misannotated genes, promiscuous enzymes, unknown reactions and pathways, as well as the elusive underground metabolism [8, 9]. Therefore, filling the knowledge gaps in the draft models is vital for comprehensive understanding of the cellular functions to drive biomedical applications [10].

Discovering the missing reactions in the draft GEMs is the core of gap-filling task. The traditional gap-filling methods are generally based on optimization, which typically require phenotypic data obtained from experiments, such as the biomass composition, to evaluate the consistency of the simulated data of the models. For instance, GrowMatch [11] and OMNI [12] leverage linear optimization techniques to choose reactions that align with network and phenotypic evidence, including experimental flux measurements or growth patterns. Similarly, MIRAGE [13] selects reactions from a database to maintain biomass production rates based on scores derived from co-expression and classification distances between the target species and species with known enzymes. It is obvious that such kind of methods is cost expensive and inefficient. Moreover, the scarcity of experimental data for non-model organisms presents a significant barrier to their widespread applicability.

In order to avoid relying on the experimental phenotype data, many innovative gap-filling methods have been proposed. For example, GapFind/GapFill [14] and its improved version FastGapFill [15] perform gap-filling by optimizing a linear score modeling of quantitative metabolic production of the system. GLOBALFIT [16] reformulates the mixed-integer linear programming problem associated with gap-filling into a more streamlined bi-level linear optimization problem, so as to pinpoint the minimal set of metabolic network modifications. Meneco [17] defines gap-filling as a qualitative combinatorial optimization problem that is then solved by using Answer Set Programming. BoostGAPFILL [18] fills and refines the gaps by using the matrix factorization based machine learning method and the integer least squares optimization method, respectively. However, the above methods have high computational complexity, resulting in poor filling performance for large-scale metabolic models.

Recently, the exploration of hypergraph structures and their integration with machine learning techniques have opened new avenues for predicting missing reactions in GEMs. Regarding GEM as a hypergraph, and a reaction in it as a hyperedge of the hypergraph, then the missing reactions discovery can be conceptualized as the hyperedge prediction of the hypergraph. Some approaches following this roadmap have been proposed. For instance, CMM [19] and its fine-tuned version C3MM [20] adopt the expectation maximization based hyperedge prediction algorithm to predict the missing reactions in biological metabolic networks. DHNE [21] models the hyperedges by vertex embeddings in heterogeneous hypergraph networks, so that the rich structural information can be preserved. Neural Hyperlink Predictor (NHP) [22] is a GCN-based framework tailored for hyperedge prediction, applicable to both undirected and directed hypergraph structures. Hyper-sagnn [23] is a self-attention graph neural network based method for hyperedge prediction across homogeneous and heterogeneous hypergraphs. It offers a novel initialization technique for vertex embeddings via autoencoder and hypergraph random walk approaches. CHESHIRE [24] combines the spectral graph convolution through Chebyshev polynomial expansion with the initial vertex features extracted by a single-layer neural network for hyperedge prediction, facilitating the inference of reaction feasibility. Nevertheless, existing methods overlook the biological specificity of metabolic reactions by treating them as mere connections between vertices. Specifically, they do not differentiate between substrates and products, neglecting their distinct properties and roles in the reactions. This oversight hampers the ability to capture subtle biological characteristics accurately, consequently affecting predictive model accuracy.

To address above mentioned issue, we propose a dual-scale fused hypergraph convolution-based hyperedge prediction model (DSHCNet) for predicting missing reactions in GEMs. In particular, DSHCNet fully considers the heterogeneity inherent in metabolic reactions and distinguishes between substrate and product vertices in vertex representation. We model a GEM as a hypergraph, representing reactions as hyperedges between substrate and product vertices. Initially, each hyperedge is modeled as a heterogeneous complete graph involving substrate–substrate, product–product, and substrate–product associations. Then we decompose this heterogeneous graph into substrate–substrate, product–product, and substrate–product subgraphs according to different types of associations. Both the substrate and product subgraphs are homogeneous complete, called homogeneous scale; the substrate-product subgraph is a heterogeneous complete bipartite, called heterogeneous scale. And then we design different graph convolution-based models for message passing among vertices to respectively extract features of the vertices at two scales, coupled with an attention mechanism for fusing the features of two scales. This process ensures that effective information exchange between substrate and product vertices, as well as among substrate or product vertices, leading to more significant feature embeddings. Finally, we use the graph pooling function to generate the representative feature of the hyperedge for reaction prediction.

In all experiments, DSHCNet demonstrated superior performance in effectively predicting missing reactions in GEMs while also improving the prediction of their metabolic phenotypes.

## Materials and methods

### Datasets

We mainly used BiGG models and universal reaction pool dataset, Fermentation metabolite dataset, and Substrate utilization dataset for the evaluation experiments in the work. Their detailed descriptions and the setting of the culture media in the experiment can be found in the Supplementary materials (Supplementary Notes SN1 and SN2).

### Description of the DSHCNet

#### Problem of reaction prediction

A draft GEM with $m$ reactions can be represented with a hypergraph $G=\left(V,E\right)$, where $V=\left\{{v}_1,{v}_2,\dots, {v}_n\right\}$ is the vertices set corresponding to all metabolites in the GEM and the reaction pool, and $E=\left\{{e}_1,{e}_2,\dots, {e}_m\right\}$ is the hyperedge set each of which corresponds to a reaction in the GEM, $n$ and $m$ are, respectively, the number of vertices and edges of $G$. Since a reaction may contain multiple metabolites (substrates and products), each edge in $E$ is a hyperedge between a set of substrates to a set of products. Therefore, predicting missing reactions in a GEM can be defined as a hyperedge (hyperlink) prediction problem of the corresponding hypergraph $G$. Without causing ambiguity, we will not differentiate between hyperedges, reactions, or hyperlinks hereinafter.

Since a hyperedge can contain more than two vertices, we simply represent a hyperedge with the subset of vertices involved in it. For example, $e=\left({v}_1,{v}_2,\dots, {v}_k\right)$ represents vertices ${v}_1,{v}_2,\dots, {v}_k$ forming the hyperedge $e$. Therefore, the hyperedge prediction problem can be delineated as follows:

Let hypergraph $G=\left(V,E\right)$ be a hypergraph, $V=\left\{{v}_1,{v}_2,\dots, {v}_n\right\}$ and $E=\left\{{e}_1,{e}_2,\dots, {e}_m\right\}$ be the sets of vertices and hyperedges in $G$, respectively. Given a threshold value $s$, we first learn a mapping function $f:P(V)\to{\mathbb{R}}^{+}$, such that for any $\left\{{v}_1,{v}_2,\dots, {v}_k\right\}\subseteq V$ :


(1) \begin{equation*} f\left({v}_1,{v}_2,\dots, {v}_k\right)\left\{\begin{array}{@{}l}\ge s, if\ \left({v}_1,{v}_2,\dots, {v}_k\right)\in E\\ <s, if\ \left({v}_1,{v}_2,\dots, {v}_k\right)\notin E\end{array}\right. \end{equation*}

where $P(V)$ is the power set of vertices set $V$. Then, the function can be used for predicting the presence of any unknown hyperedge in the hypergraph.

#### Workflow of DSHCNet

**Figure 1:**
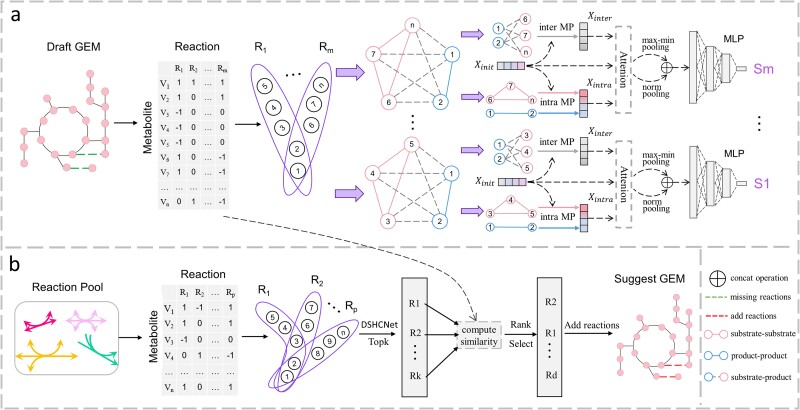
Workflow of DSHCNet. {R1, R2,…, Rm}: all metabolic reactions in the draft GEM; {R1, R2,…, Rp}: all metabolic reactions in the reaction pool; intra MP: intra-domain message passing; inter MP: inter-domain message passing. (a) Training a reaction prediction model for each GEM. (b) Predicting missing reactions in the GEM from the reaction pool.

The workflow of DSHCNet includes training and inference stages, illustrated in Fig. 1. The training stage, shown as Fig. 1(a), is to train the DSHCNet so that it learns a mapping function $f:P(V)\to{\mathbb{R}}^{+}$ for each GEM. The inference stage, shown as Fig. 1(b), is to discover the missing reactions of the GEM from the reaction pool using the trained DSHCNet model.

Since the substrates and products in a reaction have different roles, we believe it is necessary to distinguish between two different types of vertices in the hyperedge during encoding. For this purpose, in DSHCNet, each hyperedge is represented as a heterogeneous complete graph that is then decomposed into three complete subgraphs according to different kinds of associations: the substrate-product bipartite subgraph (heterogeneous), the substrate–substrate subgraph (homogeneous), and the product–product subgraph (homogeneous). Subsequently, distinct graph convolution network (GCN) models are used for the subgraphs to enable effective message passing among vertices so to extract feature embeddings for each vertex. As a result, every substrate (product) obtains two feature embeddings respectively at homogeneous and heterogeneous scales, which are then integrated as a comprehensive feature embedding of it. The hyperedge can then be represented via all feature embeddings of the vertices in it based on the graph pooling.

Based on the generated features, a multi-layer perceptron (MLP) structure is used to yield the predictive score for each hyperedge. After training, DSHCNet can be used to discover the missing reactions of the draft GEM. Specifically, DSHCNet can output the prediction score for each reaction in the reaction pool. According the scores, top-k reactions are selected as the candidates which are then evaluated according to the similarities (see Supplementary Note SN3) with the existing reactions in the draft GEM. Those are dissimilar to the existing reactions are considered as the missing reactions of the GEM.

#### Initialization of the feature embeddings of vertices

Let $G=\left(V,E\right)$ be the hypergraph of a GEM, we can initialize the feature embeddings for all of vertices based on the $H={\left\{{h}_{ij}\right\}}_{n\times m}$ of the GEM as follows:

First, we, respectively, generate the substrate and product characteristic matrices $HS={\left\{{hs}_{ij}\right\}}_{n\times m}$ and $HP={\left\{{hp}_{ij}\right\}}_{n\times m}$ as follows:


(2) \begin{equation*} {hs}_{ij}=\left\{\begin{array}{@{}l}1, if\ {h}_{ij}\le 0\\{}0, if\ {h}_{ij}>0\end{array}\kern-2.5pt,\right.\ {hp}_{ij}=\left\{\begin{array}{@{}l}1, if\ {h}_{ij}>0\\{}0, if\ {h}_{ij}\le 0\end{array}\ \right. \end{equation*}

that is, we set the positive values to 0 and negative or zero values to 1 to obtain the substrate characteristic matrix $HS$, and set all the negative or zero values to 0 and the positive values to 1 to obtain the product characteristic matrix $HP$. Therefore, we have three matrices to describe the GEM from the perspectives of reaction, substrate, and product respectively.

Then, for each matrix, we use a single-layer neural network for linear transformation to obtain a matrix with a dimension of $n\times 256$ where each row represents the embedding feature of the corresponding vertex. In all, we get three embedded matrices:


(3) \begin{equation*} {X}_H= linear(H), {X}_{HS}= linear(HS), {X}_{HP}= linear(HP) \end{equation*}

By adding three embedded matrices, we get matrix ${X}_{init}$, representing the initial feature embeddings of the vertices:


(4) \begin{equation*} {X}_{init}={X}_H+{X}_{HS}+{X}_{HP} \end{equation*}

where each row represents the feature embedding vector of a vertex.

#### Feature embeddings of hyperedges

Since a hyperedge is represented as the set of vertices, we can represent its feature embedding based on the feature embedding vectors of the vertices. Let $e=\left({v}_{s1},{v}_{s2},\dots, {v}_{sk},{v}_{p1},{v}_{p2},\dots, {v}_{pl}\right)$, the feature embedding of $e$ can then denoted as the matrix


(5) \begin{equation*} {X}_e=\left\{{x}_{s1},{x}_{s2},\dots, {x}_{sk},{x}_{p1},{x}_{p2},\dots, {x}_{pl}\right\} \end{equation*}

where each component is the feature vector of a vertex in $e$. Suppose ${V}_S=\left\{{v}_{s1},{v}_{s2},\dots, {v}_{sk}\right\}$ and ${V}_P=\{{v}_{p1}, {v}_{p2},\dots, {v}_{pl}\}$ are respectively the sets of substrate and product vertices in $e$, ${V}_{S}\cap{V}_{P}=\varnothing$ then we can further obtain the feature embeddings of the substrates and the products based on the feature vectors of the corresponding vertices:


(6) \begin{equation*} {X}_S=\left\{{x}_{s1},{x}_{s2},\dots, {x}_{sk}\right\},{X}_P=\left\{{x}_{p1},{x}_{p2},\dots, {x}_{pl}\right\} \end{equation*}

#### Massage passing between vertices

In order to obtain the comprehensive feature representations of the reactions in the GEM, we adopt two kinds of graph convolutional networks (GCNs) to perform message passing between vertices on the hyperedges at two scales, enabling sufficient message passing between vertices of the same kinds and between vertices of different kinds.

For such purpose, we first model each hyperedge as a heterogeneous complete graph with each vertex being adjacent to each other, and then decompose the graph into subgraphs at heterogeneous and homogeneous scales.

For a hyperedge $e=({v}_{s1},{v}_{s2},\dots, {v}_{sk},{v}_{p1},{v}_{p2},\dots, {v}_{pl})$, ${V}_S=\{{v}_{s1}, {v}_{s2}, \dots, {v}_{sk}\}$ and $V_P=\{v_{p1},v_{p2},\dots, v_{pl}\}$ are respectively the sets of substrate and product vertices in $e$, ${V}_{S}\cap{V}_{P}=\varnothing$. We generate a complete heterogeneous graph ${G}_e$ with $k+l$ vertices, each vertex being adjacent to each other. Subsequently, ${G}_e$ is decomposed into three subgraphs at two scales according to the different kinds of associations: substrate–substrate association graph ${G}_S$, product–product association graph ${G}_P$, and substrate–product association graph ${G}_{SP}$. Obviously, ${G}_S$, ${G}_P$ are complete and at homogeneous scale, and ${G}_{SP}$ is the complete bipartite and at heterogeneous scale, their corresponding adjacency matrices are denoted as ${A}_S$, ${A}_P$, and ${A}_{SP}$, respectively.

Since ${G}_S$ and ${G}_P$ are homogeneous, we use the GCNs [25] to achieve the homogeneous scale message passing between vertices. That is to say, message passing occurs between substrate vertices and product vertices, respectively. We refer to this kind of message passing as the intra-domain message passing. The implementation process is as follows:


(7) \begin{equation*} {X}_S= Htanh\left({D_S}^{-\frac{1}{2}}{A}_S{D_S}^{-\frac{1}{2}}{X}_S{W}_S\right) \end{equation*}

(8) \begin{equation*} {X}_P= Htanh\left({D_P}^{-\frac{1}{2}}{A}_P{D_P}^{-\frac{1}{2}}{X}_P{W}_P\right) \end{equation*}

(9) \begin{equation*} {X}_{intra}=\left[{X}_S,{X}_P\right] \end{equation*}

where ${D}_S$ and ${D}_P$ represent the degree matrices of ${A}_S$ and ${A}_P$ ; ${W}_S$ and ${W}_P$ represent the trainable weight matrices, and $Htanh\left(\cdotp \right)$ is the Hard-tanh activation function. ${X}_{intra}$ represents the homogeneous scale feature embeddings of the vertices.

Regarding the heterogeneous graph ${G}_{SP}$, it is a bipartite composed of product-substrate associations. Inspired by LightGCN [26], we specifically design a bipartite graph convolutional model to facilitate message passing between product and substrate vertices. We call this kind of message passing as the inter-domain message passing, formulated as follows:


(10) \begin{equation*} {X}_{inter}=\left[{X}_S,{X}_P\right]= Htanh\left({D}^{-\frac{1}{2}}A{D}^{-\frac{1}{2}}\left[{X}_S,{X}_P\right]{W}_{inter}\right) \end{equation*}

where


(11) \begin{equation*} A=\left[\begin{array}{@{}cc@{}}0& {A}_{SP}\\{}\ {A_{SP}}^T& 0\end{array}\right] \end{equation*}

$D$ represents the degree matrix of $A$ ; ${W}_{inter}$ represents the trainable weight matrix. ${X}_{inter}$ represents the heterogeneous scale feature embeddings of the vertices.

Now we have two feature embeddings for each vertex respectively at homogeneous and heterogeneous scales (Equations 9 and 10). We have also obtained the initial feature embeddings of the vertices according to the stoichiometric matrix $H$ (Equation 4). Therefore, we can adaptively fuse three kinds of feature embeddings through the attention mechanism to obtain the final vertex embeddings of the vertices:


(12) \begin{equation*} X={\beta}_1 linear\left({X}_{init}\right)+{\beta}_2{X}_{intra}+{\beta}_3{X}_{inter} \end{equation*}

where the attention scores (${\beta}_1$, ${\beta}_2$, ${\beta}_3$) are derived through neural network training, representing the significance of different features. This process can be implemented using a single layer of linear transformation.

#### Hyperedge prediction

Given a candidate hyperedge $e=\left({v}_1,{v}_2,\dots, {v}_k\right)$, we can obtain its characterizing features by pooling the embedding features of the vertices in it. Since the norm and maxmin pooling functions have been shown to be effective in hyperedge prediction tasks [24, 27], we also use these two pooling functions, illustrated as Equations (13) and (14), to pool the features of the vertices in DSHCNet:


(13) \begin{equation*} {y}_{norm}={\left(\frac{1}{| e|}\sum_{v_i\in e}{x}_i^2\kern0.1em \right)}^{\frac{1}{2}} \end{equation*}

(14) \begin{equation*} {y}_{maxmin}=\underset{v_i\in e}{\mathit{\max}}\kern0.1em \left\{{x}_i\right\}-\underset{v_i\in e}{\mathit{\min}}\kern0.1em \left\{{x}_i\right\} \end{equation*}

where ${x}_i$ is the embedding feature vector of vertex ${v}_i$.

The predicted score of the hyperedge $e$ can then be obtained by Equation (15):


(15) \begin{equation*} Score(e)= Sigmod\left( MLP\left({y}_{norm}\ \Big\Vert\ {y}_{maxmin}\right)\right) \end{equation*}

where || denotes the vector concatenation operation, $Sigmod\left(\cdotp \right)$ and $MLP$ represent the sigmoid activation function and MLP, respectively. Based on the predicted results, we can choose up to the top-k hyperedges from those with scores greater than 0.5 as the candidates for discovering the missing reactions in the draft GEM.

#### Training of DSHCNet

We used the following loss function during the training process:


(16) \begin{equation*} \mathcal{L}=\frac{1}{\mid E\mid}\sum_{e^{+}\in E}\sigma \left(\left(\frac{1}{| F|}\sum_{e^{-}\in F}{S}_{e^{-}}\right)-{S}_{e^{+}}\right) \end{equation*}

where $E$ is the set of positive samples, $F$ is the set of negative samples, $\sigma \left(\cdotp \right)=\mathit{\log}\left(1+\mathit{\exp}\left(\cdotp \right)\right)$ is the logistic function, and ${S}_{e^{+}}$ and ${S}_{e^{-}}$ are the prediction scores of positive samples and negative samples, respectively. The Adam optimizer [28] is used to minimize the loss function.

Additionally, we developed a two-stage strategy for generating negative samples for training (see Supplementary Note SN4). The initial learning rate of the optimizer *lr* = 0.01, the number of layers of MLP is 2, the dropout rate *μ* = 0.2, and the training epoch *r* = 35.

#### Experimental strategy and evaluation metrics

We conducted different kinds of experiments to compare our DSHCNet with the representative methods in this field, NHP [22], Hyper-sagnn [23], and CHESHIRE [24], to evaluate the performance of our method. Firstly, we evaluated their abilities of predicting known metabolic reactions of the GEMs. We call this as the internal metabolic reaction prediction experiment. Secondly, we compared their abilities of recovering missing reactions from the reaction pool. Lastly, we further compared the metabolic phenotype prediction abilities of the GEMs filled by different methods. To minimize the impact of random variation, we repeated each experiment five times and evaluated the average performance.

The area under the receiver operating characteristic curve (AUROC) and the area under the precision-recall curve (AUPR) were used as the primary evaluation metrics. We also calculated recall (RE), F1 score (F1), and accuracy (ACC) as the auxiliary evaluation metrics.

## Results

### Internal metabolic reaction prediction

**Figure 2:**
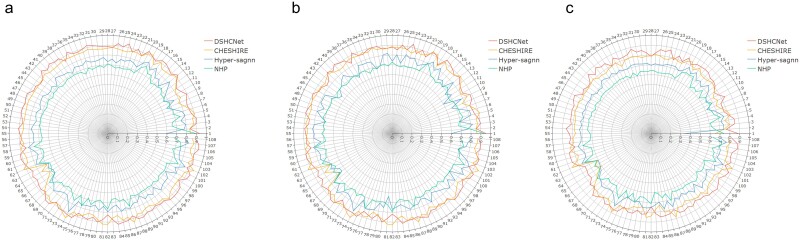
Experimental results of internal metabolic reaction prediction. (a–c) Comparison of AUROC (a), AUPR (b), and F1-Score (c) values obtained with different methods in internal metabolic reaction prediction experiments.

The experiment is described in Supplementary Note SN5. The performance across all GEMs of our DSHCNet and other three compared methods is summarized in Table 1, and the detailed performance of different methods on different GEMs is shown in Fig. 2 and also listed in Supplementary Tables S3–S5. In Table 1 and other tables below, the values displayed in bold and underlined represent the best and second best results corresponding to the respective evaluation metrics. We can see that DSHCNet has achieved the best average prediction performance across all GEMs. Moreover, from the maximum and minimum AUROC values obtained by different methods on various GEMs, it can be seen that DSHCNet exhibits a smaller range of variation, with AUROC values between 0.826 and 0.938, indicating that DSHCNet performs more stably on different GEMs than other methods. From Fig. 2 and Supplementary Tables S3–S5, we can further see that DSHCNet has more stable predictive performance on different GEMs, as well as outperforms other methods in most GEMs. The results suggest that differentiating the roles of substrates and products within the reactions can help to obtain more accurate information characterizing the reactions, thus improving the prediction performance of DSHCNet for the reactions.

**Table 1:** Comparison of predicted results for internal metabolic reactions using different methods

Methods	AUROC-max	AUROC-min	AUROC-avg	AUPR-avg	F1-avg
NHP [22]	0.898	0.644	0.697	0.710	0.648
Hyper-sagnn [23]	0.889	0.592	0.755	0.754	0.711
CHESHIRE [24]	**0.956**	0.689	0.865	0.868	0.787
DSHCNet	0.938	**0.826**	**0.894**	**0.881**	**0.830**

### Discovering missing reactions from reaction pool

Now that DSHCNet has achieved good prediction performance for the metabolic reactions in various GEMs, we further evaluated its ability to discover missed reactions of a draft GEM from the reaction pool. The experiment is described in Supplementary Note SN6.

**Figure 3:**
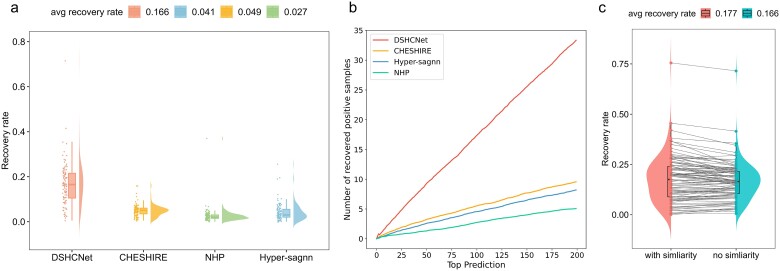
Experimental results of recovering missing reactions with reaction pools. (a) Recovery rate results of the top 200 reactions by different methods. (b) Results of the impact of different k values on the recovery rate of each method. (c) Compare the results of whether to include a similarity filtering step.

First of all, we selected the predicted top 200 reactions with the highest confidence scores for each method, and compare their top-200 recovery rates. As shown in Fig. 3(a), DSHCNet achieved an average top-200 recovery rate of 0.166 across all GEMs, surpassing the second-best method by 11.7%. This suggests DSHCNet’s superior capability in detecting missing reactions. Figure 3(b) displays that the numbers of recovered missing reactions for all methods increase with the increase of *k*, but DSHCNet consistently outperforms others at various *k* settings. This trend confirms DSHCNet’s significant advantage in recovering missing reactions. Hereinafter, we set *k* to 200 in the following experiments.

It should be noted that the strategy of solely relying on the predicted scores to identify missing reactions may be inaccurate as high-scored reactions might already be included in the GEM. Therefore, we adopted a two-round of selection strategy to identify missing reactions in our method. First, we selected the top 300 reactions with the highest confidence as candidates. Next, we calculated the distance correlation coefficients (see Supplementary Note SN3) between these reactions and the known reactions in GEM, and reordered the 300 predicted reactions in descending order of these coefficients, and selected the top 200 least similar reactions as the missing ones. As shown in Fig. 3(c), we found that the adoption of the two-round selection strategy made the improvement of the recovery rate by 1.1%, validating the effectiveness of such strategy.

### Metabolic phenotype prediction

The above experiments have verified the ability of DSHCNet to discover known missing reactions from the reaction pool. However, due to the fact that current draft GEMs themselves may be incomplete with unknown missing reactions, we are not sure whether the others of the 200 predicted reactions are unknown missing reactions of a GEM, or how many of them are unknown missing reactions. Now that metabolic reactions represent the conversion of metabolites to metabolites, it is possible to evaluate the predictive abilities of the models for missing reactions by simulating the secretion of metabolites or the consumption of substances of the GEMs. Therefore, we filled the draft GEMs with the predicted missing reactions, and then conducted the metabolic phenotype prediction experiments using the gap-filled GEMs. Specifically, we reconstructed the draft GEMs for specific organisms using CarveMe [6], denoted as GEM (Draft). Then we generated different filled-gap GEMs by adding 200 reactions predicted/selected by different methods. Accordingly, we denoted the GEMs filled with top reactions predicted by DSHCNet, NHP [22], Hyper-sagnn [23], and CHESHIRE [24] as GEM (DSHCNet), GEM (NHP), GEM (Hyper-sagnn), and GEM (CHESHIRE), respectively. We also generated another kind of gap-filled GEMs, denoted as GEM (Random), by adding the randomly selected 200 reactions from the reaction pool to the GEM (Draft). As a result, we compared the metabolic phenotype prediction abilities of six different kinds of GEMs in the following two experiments.

### Prediction of fermentation products

The experiment is described in Supplementary Note SN7. Table 2 shows the performance of fermentation products predictions across 24 filled-GEMs using various methods. In which we can see that GEM (DSHCNet) is superior to other GEMs on most metrics except for ACC. We noticed that GEM (DSHCNet) did not reach comparable ACC scores than most of other GEMs, even lower than that either of GEM (Draft) or GEM (Random). This discrepancy is largely due to the severe imbalance between positive and negative samples in the data, making ACC an unreliable indicator of model performance. Instead, AUPR and F1 scores, which GEM (DSHCNet) leads in, offer more objective measures of the model’s predictive ability, indicating that DSHCNet can effectively fill the gaps in the draft GEMs.

**Table 2:** Comparison of phenotype prediction results from different methods

GEM	AUPR	AUROC	F1	ACC	RE
GEM (Draft)	0.384	0.502	0.253	0.612	0.213
GEM (Random)	0.451	0.535	0.337	0.614	0.304
GEM (CHESHIRE)	0.534	0.587	0.450	0.625	0.482
GEM (NHP)	0.443	0.534	0.316	**0.631**	0.268
GEM (Hyper-sagnn)	0.421	0.513	0.300	0.602	0.268
GEM (DSHCNet)	**0.606**	**0.632**	**0.524**	0.605	**0.702**

**Figure 4:**
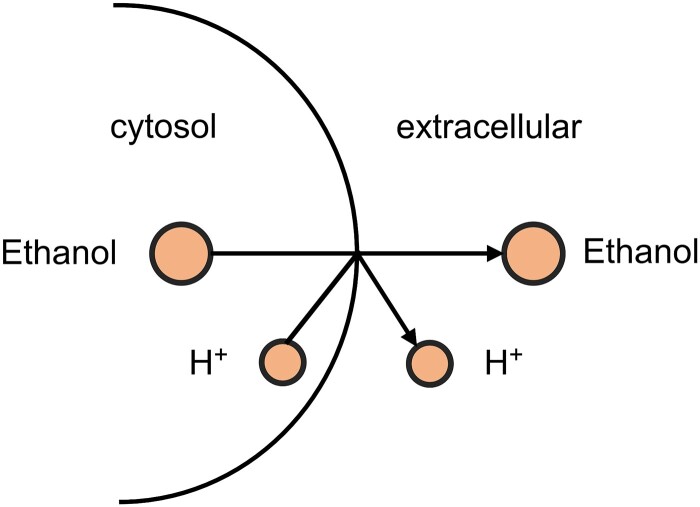
Visualization of the ethanol transport reaction.

Now that GEM (DSHCNet) achieves the best prediction results, we further utilized the method same as [24] to find which reaction(s) in the top 200 selected ones that fill the gaps, in order to understand how our method fills the GEMs better than the others. For instance, GEM (DSHCNet) introduced a single ethanol transport reaction in the *Clostridium perfringens* model, resulting in the extracellular production of ethanol (see Fig. 4), which corrects the false negative predictions of both GEM (Draft) and GEM (CHESHIRE). Furthermore, GEM (DSHCNet) can also correct the false positive predictions of other models. For example, both GEM (Draft) and GEM (CHESHIRE) predicted the lactate production, which contradicted experimental results. However, GEM (DSHCNet) incorporated a reaction pathway that converts citric acid into acetic acid and oxaloacetic acid in the *Clostridium butyricum* model that is then able to convert lactic acid and acetic acid into butyrate [29], demonstrating that the reactions introduced by DSHCNet could potentially alter the conversion of intermediary metabolites, consequently impacting the lactate production.

### Substrate utilization test

**Figure 5:**
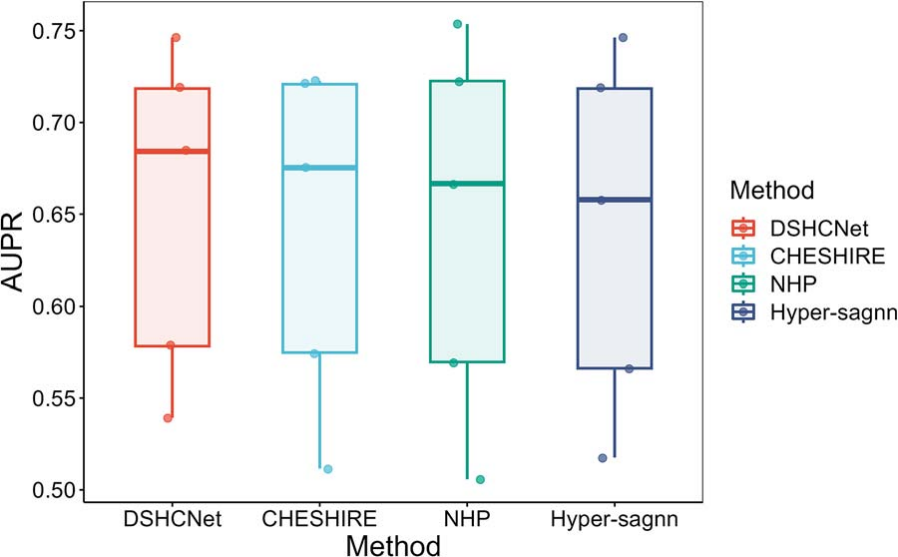
Comparison of AUPR results in substrate utilization experiments for different methods.

In addition to evaluating the gap filling ability of metabolite secretion, we also validated the effectiveness of DSHCNet in filling gaps of substrate utilization for growth. This experiment is described in Supplementary Note SN8. As shown in Fig. 5, DSHCNet demonstrates a better AUPR performance than the other four methods.

### Ablation study

To verify the efficacy of each component in DSHCNet, we conducted an ablation experiment. The dataset and evaluation process used in this experiment were the same as those in the prediction of fermentation products experiment (Supplementary Note SN7). We started by removing the heterogeneity of vertices, including both the division of heterogeneous graphs and the initialization of vertex feature heterogeneity, and employed a single-layer GCN as the feature extraction module to establish a baseline comparison model. Subsequently, we assessed the influence of various components by either adding or deleting them. From the experimental results in Table 3, it was observed that the addition of heterogeneous graph division and the initialization of vertex feature heterogeneity, significantly improved the model’s effectiveness. This improvement was evidenced by an increase in AUPR from 0.539 to 0.583, underscoring the importance of distinguishing heterogeneity among vertices. Furthermore, incorporating an attention mechanism further enhanced both AUPR and AUROC, indicating its effectiveness in integrating information across domains, thus boosting the model’s learning capabilities.

**Table 3:** Result of ablation experiment.

Methods	AUPR	AUROC	F1	ACC	RE
Single-layer GCN	0.539	0.598	0.450	**0.653**	0.446
No feature initialization	0.556	0.582	0.471	0.580	0.589
No attention	0.583	0.605	0.500	0.591	0.643
DSHCNet	**0.606**	**0.632**	**0.524**	0.605	**0.702**

## Conclusion

In this paper, we propose DSHCNet, a hyperedge prediction model utilizing dual-scale hypergraph convolution, applied for predicting missing reactions in GEMs. Distinct from existing methods, DSHCNet preserves the heterogeneity of hyperedges by distinguishing between substrate and product vertices. It constructs a heterogeneous graph network reflecting this heterogeneity and builds two distinct graph convolution-based models to enable message passing at both homogeneous and heterogeneous graph scales. Additionally, it employs attention mechanisms for effective feature fusion. Comparative experiments with other state-of-the-art hyperedge prediction models demonstrate that DSHCNet enhances the prediction capability of hyperedges through dual-scale hypergraph convolution, effectively predicting missing reactions in GEMs. Importantly, DSHCNet exhibits superior performance in more biologically meaningful phenotype prediction experiments, adeptly filling gaps in draft GEMs and markedly surpassing competing methodologies. Additionally, we enhance the interpretability of test results by looking for key responses that lead to phenotypic changes.

Despite DSHCNet achieving satisfactory performance, its effectiveness is somewhat constrained by the existing reaction pools. Besides, the variability in the number and types of reactions across different databases poses a significant challenge to model adaptation. To overcome these obstacles, our future efforts will focus on interconnecting diverse databases, with the goal of constructing a more comprehensive database that can significantly advance the field of metabolomics.

**Key Points**

- We propose a model called DSHCNet, a hyperedge prediction model based on dual-scale fused hypergraph convolution, to predict missing reactions in GEMs.
- We have preserved the heterogeneity of hyperedges, distinguishing substrate and product vertices not only based on the feature level but also constructing a heterogeneous graph network according to different types of connections, which enhances message passing between vertices within a hyperedge.
- We build corresponding graph message passing models at two scales (homogeneous graph scale and heterogeneous graph scale) to extract features in their respective domains, then fully integrate them through the attention mechanism, and, finally, employ MLP, combined with graph pooling operations, to obtain more representative hyperedge embeddings.
- DSHCNet maintains a stable performance in comprehensive experiments and significantly outperforms other methods. Particularly in phenotype prediction, it improves the prediction of multiple phenotypes for various GEMs without requiring the input of phenotype data, excelling in the task of phenotype prediction.

## Supplementary Material

Supplementary_materials_bbae383

Supplementary_Table_S3-S5_bbae383

## Data Availability

The implementation of DSHCNet and the preprocessed data is available at: https://github.com/1075793472/DSHCNet.
