# Supplementary material for: A dual-scale fused hypergraph convolution-based hyperedge prediction model for predicting missing reactions in genome-scale metabolic networks
Source: Brief Bioinform. 2024 Aug 5;25(5):bbae383. doi: 10.1093/bib/bbae383 (PMC11299038; doi:10.1093/bib/bbae383)
Supplement: Supplementary_materials_bbae383 [file supplementary_materials_bbae383.docx]

Supplementary Materials:

A dual-scale fused hypergraph convolution-based hyperedge prediction model for predicting missing reactions in genome-scale metabolic networks

Weihong Huang, Feng Yang, Qiang Zhang, Juan Liu *

School of Computer Science, Wuhan University, Wuhan, Hubei, 430072, China,

1.Supplementary Notes

## Supplementary Note SN1: Description of datasets

**BiGG models and** **universal reaction pool**

The 108 BiGG models and the universal reaction pool used in this work were downloaded from the BiGG database [1] (http://BiGG.ucsd.edu). We adopted the same strategies as [2] to preprocess the reactions in the BiGG models and the reaction pool. For each model, we removed the biomass, exchange, demand and sink reactions, and the reactions involving compartments other than cytosol, periplasm, and extracellular space. For the universal reaction pool, we eliminated reactions with empty names and excluded reactions whose identifiers start with letter “r” and follow by digital numbers, for all of which are derived from non-microbial GEMs. As a result, there are 10,393 metabolites (unique IDs) and 16,337 reactions (unique IDs) remained for our reaction prediction experiments.

**Fermentation metabolite dataset**

The Fermentation metabolite dataset was derived from the study in [3]. It contains 24 bacterial genomes (Supplementary Table S1) along with measurements of 8 fermentation products (acetic acid, butyric acid, ethanol, formic acid, lactic acid, butanol, propionic acid, succinic acid) in the culture media. The compositions of the culture media are detailed in the Supplementary materials. This dataset is used for the metabolic phenotype prediction experiment.

**Substrate utilization dataset**

Substrate utilization dataset comes from [4], which is also used for the metabolic phenotype prediction experiment of our work. The dataset was derived from the experimental tests on the growth of 5 bacterial species using the Biolog Phenotypic Array [5]. Except for the nitrogen source testing for Pseudomonas aeruginosa PAO1, both carbon and nitrogen source utilizations were assessed for all 5 species (Supplementary Table S2). Additionally, further tests were conducted to evaluate the utilization of phosphorus and sulfur sources by E. coli str. K-12 substr. MG1655, B. subtilis 168, and R. solanacearum GMI1000. The compositions of the culture media are detailed in the Supplementary materials.

## Supplementary Note SN2: Culture media settings

**Fermentation metabolite dataset**

In phenotype testing, we followed the cultivation setup proposed by Zimmermann et al. [3], where the specific composition of the medium and the maximum allowable fluxes can be obtained from <https://github.com/Waschina/gapseqEval>.

**Substrate utilization dataset**

The substrate utilization experiments were conducted using media compositions available from https://github.com/cdanielmachado/carveme, with GEMs constrained within the same minimal M9 medium. The default sources for carbon, nitrogen, sulfur, and phosphorus are glucose, ammonia, sulfate, and phosphate, respectively. Specifically, for Shewanella oneidensis, the default carbon source is DL-lactate. To simulate growth on each substrate in the Biolog array, the default sources for the same type of substrate (i.e., carbon, nitrogen, sulfur, and phosphorus) in the minimal M9 medium were replaced with the respective substrates. The maximum uptake rate for all Biolog substrates was set at 10 mmol/gDW/h, while all other compounds in the M9 medium were unrestricted.

## Supplementary Note SN3: Discovery of missing reactions via similarity

The selected candidates are considered to correspond to the reactions that are most likely in the GEM. However, it is unclear which of them are already known reactions in the GEM draft whereas the others are missing reactions that should be identified out. Therefore, it is necessary to exclude the known reactions and explore the missing reactions that should be filled into the GEM draft. For such purpose, we employ the distance correlation coefficient to measure the correlations between the predicted reactions and the known reactions in the GEM draft. Let $R_{predict}$ and $R_{known}$ be the predicted and known reactions respectively, their distance correlation coefficient can be calculated as follows:

$$DCor(R_{predict},R_{known})=\frac{dCov(R_{predict}, R_{known})}{\sqrt{dVar(R_{predict})\cdot dVar(R_{known})}}$$

where ${dCov}^{2}\left( R_{predict},R_{known} \right)$ is the distance covariance between $R_{predict}$ and $R_{known}$; ${{dVar}^{2}(R_{predict})=dCov}^{2}\left( R_{predict},R_{predict} \right)$ and ${{dVar}^{2}(R_{known})=dCov}^{2}\left( R_{known},R_{known} \right)$ are respectively the variances of $R_{predict}$ and $R_{known}$. It is obvious that $DCor\left( R_{predict},R_{known} \right)\in[0,1]$; and the larger it is, the stronger the correlation between $R_{predict}$ and $R_{known}$; when it is zero, $R_{predict}$ and $R_{known}$ are independent of each other.

For a candidate (predicted) reaction, we calculate its distance correlation coefficients with all known reactions in the GEM draft. Only when its correlation with any known reaction is less than a threshold, it is considered to be a missing reaction that needs to be filled in the GEM draft.

## Supplementary Note SN4: Generation of negative samples

For each GEM draft, we train the DSHCNet to obtain a hyperedge prediction model for discovering the missing reactions from the reaction pool. However, we have only the positive samples coming from existed reactions in the GEM draft, lacking the negative samples. In order to generate the negative samples that are unlikely the missing reactions of the GEM, we propose a two-step random replacement strategy to generate the negative samples in a 1:1 ratio to positive samples. Firstly, randomly replacing half of the metabolites in an existing reaction (positive sample) with metabolites randomly selected from the reaction pool to get an immediate sample. Then, we randomly select half of the metabolites in the immediate sample and change their types, that is, changing original substrates into products, and changing original products to substrates. By this way, we obtain a negative sample from each positive sample.

## Supplementary Note SN5: Experiment for internal metabolic reaction prediction

We used the 108 high-quality GEMs in the BiGG database (Supplementary Note SN1) for this experiment. For each GEM, we randomly selected 60% of the reactions as the positive training data and the remaining 40% as the positive testing data. For our DSHCNet method, we generated the negative samples using above mentioned two-step strategy (Supplementary Note SN4). For other three compared methods, since they did not distinguish between product vertices or substrate vertices, we simply adopted the first step to generate the negative samples, as their authors did in the original papers.

## Supplementary Note SN6: Experiment for discovering missing reactions

We used the 108 high-quality GEMs and the universal reaction pool in the BiGG database (Supplementary Note SN1) for this experiment. It is difficult to directly evaluate the ability of different models to discover missed reactions, since there is lack of prior knowledge about which reactions belong in the GEM but are missing. Therefore, in this experiment, we randomly removed 10% reactions from the GEM and put them into the reaction pool to get an extended reaction pool (containing approximately 17,000 reactions). The remaining 90% reactions were used to train the models that were then used to predict each reaction in the extended reaction pool. Since we have already put the 10% known reactions of the GEM in the extended reaction pool, we can indirectly evaluate the ability of discovering missing reactions of a methods via the recovery rate of the 10% known missed reactions.

In this paper, we used the top-*k* recovery rate to evaluate the missed reactions discovery ability of the method. The top-*k* recovery rate refers to the ratio of known missing reactions among the top *k* selected reactions to all known missing reactions. In this experiment, we set *k* = 200. That is, we compared the top-200 recovery rates of the 10% known missing reactions of different methods.

## Supplementary Note SN7: Experiment for fermentation products prediction

The fermentation metabolite test data (Supplementary Notes SN1 and SN2) was used in this experiment. Following the strategy proposed by Chen et al. [2], we used PFBA [6] and FVA [7] to calculate the efflux values of the secreted metabolites. Those metabolites with efflux rates (secretion flux relative to biomass) above 10^-5^ were considered as produced by the GEM. We then compared and analyzed the model predictions against the observed data, using the observed phenotypes as positive samples and the unobserved phenotypes as negative samples.

## Supplementary Note SN8: Experiment for substrate utilization test

Substrate utilization test data (Supplementary Notes SN1 and SN2) was used in this experiment. Phenotype array simulation involved constraining each model to M9 minimal medium (with maximum uptake rates of 10 mmol/gDW/h for each compound). For each array type (carbon, nitrogen, sulfur, phosphorus), the default source of a given element (glucose, ammonia, sulfate, and phosphate) was iteratively replaced with the corresponding compound in the array. For instance, for Shewanella oneidensis, the default carbon source was DL-lactate. The filled models were analyzed by FBA, and a growth phenotype was considered feasible if the growth rate was at least 0.01 h^-1^, where experimental data for validation were obtained from [8-11].

**Supplementary references**

1. Norsigian CJ, Pusarla N, McConn JL, et al. BiGG Models 2020: Multi-strain genome-scale models and expansion across the phylogenetic tree. Nucleic Acids Res. 2020; 48: D402–D406.

2. Chen C, Liao C, Liu YY. Teasing out missing reactions in genome-scale metabolic networks through hypergraph learning. Nat. Commun. 2023; 14:1–11.

3. Zimmermann J, Kaleta C, Waschina S. Gapseq: Informed Prediction of Bacterial Metabolic Pathways and Reconstruction of Accurate Metabolic Models. Genome Biol. 2021; 22:1–35.

4. Machado D, Andrejev S, Tramontano M, et al. Fast automated reconstruction of genome-scale metabolic models for microbial species and communities. Nucleic Acids Res. 2018; 46:7542–7553.

5. Karp PD, Latendresse M, Paley SM, et al. Pathway tools version 19.0 update: Software for pathway/genome informatics and systems biology. Brief. Bioinform. 2016; 17:877–890.

6. Lewis NE, Hixson KK, Conrad TM, et al. Omic data from evolved E. coli are consistent with computed optimal growth from genome-scale models. Mol. Syst. Biol. 2010; 6.

7. Gudmundsson S, Thiele I. Computationally efficient flux variability analysis. BMC Bioinformatics 2010; 11:2–4.

8. Oh YK, Palsson BO, Park SM, et al. Genome-scale reconstruction of metabolic network in Bacillus subtilis based on high-throughput phenotyping and gene essentiality data. J. Biol. Chem. 2007; 282:28791–28799

9. Oberhardt MA, Puchałka J, Fryer KE, et al. Genome-scale metabolic network analysis of the opportunistic pathogen Pseudomonas aeruginosa PAO1. J. Bacteriol. 2008; 190:2790–2803

10. Peyraud R, Cottret L, Marmiesse L, et al. A Resource Allocation Trade-Off between Virulence and Proliferation Drives Metabolic Versatility in the Plant Pathogen Ralstonia solanacearum. PLoS Pathog. 2016; 12:1–25.

11. Price MN, Wetmore KM, Waters RJ, et al. Mutant phenotypes for thousands of bacterial genes of unknown function. Nature 2018; 557:503–509.

2.Supplementary Tables

**Supplementary Table S1**. Bacterial genomes used in our external validation for testing fermentation products.

| NCBI Assembly | Taxonomy |
| --- | --- |
| GCF_000005845.2 | Escherichia coli str. K-12 substr. MG1655 |
| GCF_000008345.1 | Cutibacterium acnes KPA171202 |
| GCF_000008545.1 | Thermotoga maritima MSB8 |
| GCF_000008765.1 | Clostridium acetobutylicum ATCC 824 |
| GCF_000011065.1 | Bacteroides thetaiotaomicron VPI-5482 |
| GCF_000011985.1 | Lactobacillus acidophilus NCFM |
| GCF_000013285.1 | Clostridium perfringens ATCC 13124 |
| GCF_000020425.1 | Bifidobacterium longum subsp. infantis ATCC 15697 |
| GCF_000020605.1 | Eubacterium rectale ATCC 33656 |
| GCF_000022965.1 | Bifidobacterium animalis subsp. lactis DSM 10140 |
| GCF_000025885.1 | Aminobacterium colombiense DSM 12261 |
| GCF.000056065.1 | Lactobacillus delbruecki subsp. bulgaricus ATCC 11842 |
| GCF_000143845.1 | Olsenella uli DSM 7084 |
| GCF_000144405.1 | Prevotella melaninogenica ATCC 25845 |
| GCF_000160535.1 | Prevotella bergensis DSM 17361 |
| GCF_000173975.1 | Anaerobutyricum halli DSM 3353 |
| GCF_000175255.2 | Zymomonas mobilis subsp. mobilis ATCC 10988 |
| GCF_000389635.1 | Clostridium pasteurianum BC1 |
| GCF_000392875.1 | Enterococcus faecalis ATCC 19433 |
| GCF_000005845.2 | Eubacterium ramulus ATCC 29099 |
| GCF_000008345.1 | Clostridium butyricum KNU-L09 |
| GCF_000008545.1 | Anaerotignum propionicum DSM 1682 |
| GCF_000008765.1 | Faecalibacterium prausnitzii A2-165 |
| GCF_000011065.1 | Escherichia coli str. K-12 substr. MG1655 |

**Supplementary Table S2.** Bacterial genomes used in our substrate utilization test data

| NCBI Assembly | Taxonomy |
| --- | --- |
| GCF_000005845.2 | Escherichia coli str. K-12 substr. MG1655 |
| GCF_000009045.1 | Bacillus subtilis 168 |
| GCF_000006765.1 | Pseudomonas aeruginosa PAO1 |
| GCF_000009125.1 | Ralstonia solanacearum GMI1000 |
| GCF_000146165.2 | Shewanella oneidensis MR-1 |
| GCF_000027325.1 | Mycoplasmoides genitalium G-37 |
